# Exosomes derived from mesenchymal stem cells improved core symptoms of genetically modified mouse model of autism Shank3B

**DOI:** 10.1186/s13229-020-00366-x

**Published:** 2020-08-17

**Authors:** N. Perets, O. Oron, S. Herman, E. Elliott, D. Offen

**Affiliations:** 1 Sagol School of Neuroscience, Tel Aviv University, Tel Aviv, Israel; 2 Sacklar School of Medicine, Tel Aviv University, Tel Aviv, Israel; 3 Faculty of Medicine, Bar Ilan University, Tzfat, Israel

## Abstract

**Background:**

Partial or an entire deletion of *SHANK3* are considered as major drivers in the Phelan–McDermid syndrome, in which 75% of patients are diagnosed with autism spectrum disorder (ASD). During the recent years, there was an increasing interest in stem cell therapy in ASD, and specifically, mesenchymal stem cells (MSC). Moreover, it has been suggested that the therapeutic effect of the MSC is mediated mainly via the secretion of small extracellular vesicle that contains important molecular information of the cell and are used for cell-to-cell communication. Within the fraction of the extracellular vesicles, exosomes were highlighted as the most effective ones to convey the therapeutic effect.

**Methods:**

Exosomes derived from MSC (MSC-exo) were purified, characterized, and given via intranasal administration to Shank3B KO mice (in the concentration of 10^7^ particles/ml). Three weeks post treatment, the mice were tested for behavioral scoring, and their results were compared with saline-treated control and their wild-type littermates.

**Results:**

Intranasal treatment with MSC-exo improves the social behavior deficit in multiple paradigms, increases vocalization, and reduces repetitive behaviors. We also observed an increase of *GABARB1* in the prefrontal cortex.

**Conclusions:**

Herein, we hypothesized that MSC-exo would have a direct beneficial effect on the behavioral autistic-like phenotype of the genetically modified Shank3B KO mouse model of autism. Taken together, our data indicate that intranasal treatment with MSC-exo improves the core ASD-like deficits of this mouse model of autism and therefore has the potential to treat ASD patients carrying the Shank3 mutation.

## Introduction

Autism spectrum disorder (ASD) is a neurodevelopmental disorder defined by social–communicational deficits, repetitive behaviors, and restricted interests. In the last two decades, ASD's etiology has been shown to be extremely complex, composed of both genetic and epigenetic variations [1–3]. Further studies have shown that this complexity translates to multiple perturbed molecular pathways [4–6]. This complexity may explain the great difficulty in finding pharmacological therapies that can reverse or ameliorate the core symptoms of ASD efficiently and across the spectrum [7]. The current approved pharmacological treatments target the comorbid behaviors frequently observed in ASD such as anxiety, hyperactivity, and impulsive-related behaviors [7, 8]. However, it seems that the greater challenge is finding a treatment that will address a combination of the core autistic behaviors, including social–communicational and repetitive/restricted interests.

In our previous study, we have shown that intraventricular administration of mesenchymal stem cells (MSC) resulted in the amelioration of the core ASD-like symptoms in the BTBR T+tf/J (BTBR) autism mouse model, including significant improvement in social interactions, maternal behavior, reduction in repetitive behaviors, and reduction in cognitive rigidity [9, 10]. Surprisingly, the ameliorating effect of transplantation of MSC in BTBR mice lasted for at least 6 months after the treatment [11]. Since it is likely that the MSCs did not survive in the transplanted tissue longer than a few weeks, we assumed that the MSCs left a long-lasting “fingerprint” via their paracrine secretion. This hypothesis was supported by several studies demonstrating that MSCs can leave long-lasting effects after transplantation by secretion of exosomes [11]. Exosomes, which are lipid nano-vesicles, which carry proteins, RNA, and miRNA, are found to be responsible for some of the intercellular communication [12, 13].

Indeed, using the same BTBR model determined that intranasal administration of human MSC-derived exosomes (MSC-exo) resulted in significant improvement in the core symptoms including social interaction, ultrasonic communication, and repetitive behaviors [14]. Furthermore, we demonstrated that MSC-derived exosomes migrate to specific neuropathological locations in rodent models for stroke, Parkinson's disease, Alzheimer’s disease (AD), spinal cord injury, and ASD. Interestingly, in the BTBR ASD model, the MSC-exo migrated to the frontal cortex and cerebellum and were taken up by neurons [15–18].

The BTBR model is an idiopathic model of ASD without a known genetic mutation that might lead to the ASD-like symptoms [19–23]. To investigate whether exosome administration will be effective also in a transgenic ASD model with a specific mutation associated with the disorder, we chose to study the Shank3B KO model [24, 25]. *SHANK3* is an essential scaffolding protein found specifically in the postsynaptic density (PSD) of excitatory neurons. Genetic variations in *SHANK3* have been shown to affect dendritic spine development [26] and reduce ionotropic and metabotropic receptor signaling [27, 28]. In addition, numerous studies have demonstrated that transgenic mice harboring mutations in *shank3* exhibit robust ASD-like behavioral phenotypes [29, 30].

We assume that the use of MSC-exo can lead to significant amelioration of the core symptoms of the genetically modified Shank3B mice model of autism. It has been demonstrated that MSC-exo can improve both pathological and cognitive functions of genetically impaired mouse models such as the 5xFAD and 3xFAD Alzheimer’s models [31–33]. Therefore, there may be an interesting interaction between the capacity of the MSC-exo which includes proteins and RNAs such as growth factors and immune-regulation proteins and RNA [34–36] and the functions of the brain cells. Meaning, although the mice have a genetic mutation, the treatment of MSC-exo may lead to alterations in gene expression and protein function, leading to cognitive and behavioral benefits.

In this line of thinking, we examined both the social and cognitive abilities of Shank3B KO mice as well as the expression of selected genes. Therefore, we performed behavioral experiments on this transgenic mouse model which included testing for social interaction, grooming, and ultrasonic vocalization (UV). Additionally, we previously showed that MSC-exo rescues ASD-like behavior in the BTBR model, and so were interested in comparing its effect on the Shank3B model as well. We also chose several inflammatory RNA markers (TNF훼, IL-1, and IBA-1) to monitor after the MSC-exo treatment, as their expression changes in BTBR due to MSC-exo treatment; however, we did not see the same effect with Shank3B.

One of the most common neurological theories for ASD symptoms is focused on the alterations in excitatory–inhibitory balance in the brain [37–39]. This theory was based on the postmortem analysis of ASD patients presenting downregulation in GABA_A_ receptors [40]. Therefore, we aimed to figure if the MSC-exo treatment may lead to changes in the inhibitory receptors of the Shank3B KO mice that may lead to the amelioration of the behavioral phenotype. We found, delicate, yet significant increase in the GABA Ra1 RNA expression in the PFC of treated Shank3B KO mice. Moreover, as we previously described, there was a difference in the evacuation timeline and pattern of the MSC-exo from the brains of pathological Shank3B KO mice in comparison to their wild-type (WT) littermates. While the MSC-exo remained in the brain 96 h post the intranasal administration in the pathological mice, they were gone from the healthy brains. Most importantly, we observed significant amelioration in the behavioral core symptoms of the treated Shank3B KO mice, compared to their saline-treated littermates, making their behavior closer to their WT littermates.

Altogether, our data suggest that exosomes can be used as a clinical candidate to ameliorate the ASD-like symptoms of the Phelan–McDermid syndrome.

## Methods

### Animal care

Mice were housed according to the Federation of Laboratory Animal Science Associations (FELASA) guidelines. All mice were bred and maintained in a vivarium at 22 °C in a 12-hr light/dark cycle, with food and water available ad libitum. The Shank3B KO line was purchased from Jackson Laboratories. Shank3B KO, and wild-type littermate mice were produced through crosses of heterozygote males and females. The genetic background for the Shank3B mouse lines is C57BL/6J. Experiments were performed with 8- to 10-week-old male mice. All experimental protocols were approved by the Animal Care and Use Committee of Bar Ilan University.

### Genotyping

To determine the *Shank3B* genotypes, DNA was extracted from ear samples notched at the time of weaning using the KAPA mouse genotyping kit. The following primers were used to determine *Shank3B* mice genotype: common Fw 5'-GAGCTCTACTCCCTTAGGACTT-3'; Rv mutant 5'-TCAGGGTTATTGTCTCATGAGC-3' (~330 bp) and for wild type: Rv 5'- TCCCCCTTTCACTGGACACCC-3' (~250 bp).

### Behavioral tests

#### Reciprocal dyadic social interaction test

The reciprocal dyadic social interaction test was done as previously described [9, 14]. Prior to the test, each mouse was separated for social isolation of 1-2 h. A five-week-old male RCF white stranger mouse was used as the social stimulus. Both the stranger and the tested mouse were placed in a 40 × 40 × 20-cm cage. During the interaction, the mice were recorded for 20 min, with the last 10 min quantified by an observer blind to treatment. Cowlog V3 software was used to score the social contact initiated by the test mouse (Helsinki University, Helsinki, Finland).

#### Three-chambered social interaction test

The test took place in a non-glare perspex box (60 × 40 cm) with two partitions that divide the box to three chambers: left, center, and right (20 × 40 cm). The mouse is placed in the middle chamber for habituation (5 min) when the entry for both side chambers is barred. Test mouse was then allowed to explore the whole arena (10 min), where they freely choose between interacting with a novel mouse in one chamber, or stay in an empty chamber (social test). After 10 min ended, a second stranger mouse is introduced to the empty chamber, and the test mouse is allowed 10 min to freely choose between interacting with the novel or familiar mouse.

#### Ultrasonic vocalizations

The ultrasonic vocalization test was done as previously described [10, 14]. Both Shank3B KO and WT males met WT females, all sexually naive. Prior to the test, each mouse was placed in separate cages for social isolation for 12 h, the female was placed in the cage of the male. UVs were recorded for the first 5 min of encounter to prevent extremely high sexual arousal and mating behaviors. The females were in the same cage in order to synchronize their estrus cycle and had met the males on the same day. UVs were recorded with the Avisoft-RECORDER v. 4.2.21 recording program. The settings included a sampling rate of 250 kHz and a format of 16 bit. For spectrogram generation, recordings were transferred to Avisoft-SASLab Pro Version 5.2.07, and a fast Fourier transformation (FFT) was conducted. Spectrograms were generated with an FFT length of 256 points and a time window overlap of 50% (100% Frame, FlatTop window).

#### Mesenchymal stem cell preparation

Human MSCs were purchased from Lonza (cat: PT-2501, Basel, Switzerland) and were cultured as previously described [35]. Before the exosome collection, the cells were cultured in exosome-free platelets medium for 3 days, and this medium was then collected.

#### Exosome purification protocol

Purification of exosomes was done using differential centrifugation protocol. First, the conditioned medium was centrifuged for 10 min at 300 g. The supernatant was recovered and centrifuged for 10 min at 2000 g. Once again, the supernatant was centrifuged for 30 min at 10,000 g. The supernatant was filtrated through a 0.22-μm filter and centrifuged for 70 min at 100,000 g. The pellet containing the exosomes and proteins was washed in phosphate-buffered saline (PBS) and then centrifuged for 70 min at 100,000 × *g*. The pellet containing the purified exosomes was resuspended in 200 μm of sterilized PBS. MSC-exo were characterized using Nanosight technology, TEM, and western blotting as previously described [14–16, 41].

### In vivo treatment protocol

At the age of 4 weeks, 10 Shank3B KO male mice were treated with a total of 20 μl MSC-exo at a concentration of 10^7^ particles/μl. The treatment was performed as previously described [14]. In general, each Shank3B KO-treated mouse received 5 μl of exosomes via intranasal administration for 4 days, every other day (altogether, 8 days of treatment). The behavioral experiment was done 3 weeks after the last treatment.

#### FACS analysis of exosomes

For FACS analysis, exosomes were coated onto 4-μm-diameter aldehyde/sulfate latex beads. Fifty microliters of exosomes were incubated with 12.5 μl of 4-μm-diameter aldehyde/sulfate latex beads (cat# A37304, Invitrogen) for 15 min at room temperature. Seven hundred mircoliters of sterile PBS was added, and the mixture was then transferred to 4 °C and gentle shaking overnight. After centrifugation, the pellet was blocked by incubation with 200 μl of 100-mM glycine for 30 min at room temperature. Exosome-coated beads were washed in PBS and resuspended in 100-μl sterile PBS. Afterwards, beads were incubated with CD63-APC (cat# 130-118-078, Miltenyi biotec), CD81-APC (cat# 130-119-787 Miltenyi biotec), or IgG1 Isotype control (cat# 130-113-434, Miltenyi biotec) fluorescent Abs for 15 min on ice in the dark. Beads were analyzed by flow cytometry using Gallios flow analyzer FACS (Beckman Coulter). Data were analyzed using the Kaluza Analysis Software (Beckman Coulter).

#### Exosome labeling

Exosomes were labeled with PKH26 (Sigma-Aldrich). PKH26 (2 μl) in 500-μl diluent was then added to 50 μl exosomes in PBS for 5 min of incubation. Exosomes were suspended in 70-ml PBS and were centrifuged for 90 min at 100,000 × *g* at 4 °C. The pellet was suspended in 200 μl of PBS [15, 16].

#### Proteomic analysis of MSC-exo

The samples were subjected to lysis and in solution tryptic digestion using the S-Trap method (by Protifi). The resulting peptides were analyzed using nanoflow liquid chromatography (nanoAcquity) coupled to high resolution, high mass accuracy mass spectrometry (Q Exactive HF). Each sample was analyzed in the instrument separately in a random order in discovery mode, and the DATA processing was done by MaxQuant v1.6.0.16. The data was searched with the Andromeda search engine against the human proteome database appended with common lab protein contaminants and the following modifications: fixed modification (cysteine carbamidomethylation) and variable modifications (methionine oxidation, asparagine and glutamine deamidation, and protein N-terminal acetylation). The quantitative comparisons were calculated using Perseus v1.6.0.7. Decoy hits were filtered out. The resulting peptides were analyzed using nanoflow liquid chromatography (nanoAcquity) coupled to high-resolution, high mass spectrometry accuracy (Q Exactive HF). Each sample was analyzed on the instrument separately in a random order in discovery mode. Raw data was processed with MaxQuant v1.6.0.16. The data was searched with the Andromeda search engine against the human proteome database appended with common lab protein contaminants and the following modifications: fixed modification (cysteine carbamidomethylation) and variable modifications (methionine oxidation, asparagine and glutamine deamidation, and protein N-terminal acetylation). The quantitative comparisons were calculated using Perseus v1.6.0.7. Decoy hits were filtered out. Gene onthology was performed by using the ToppGene Suite [42]. Presented GO terms met a *p* value of < 0.05 at Benjamini–Yekutieli false detection rate (FDR).

#### Ex vivo imaging

For immunostaining, Shank3B KO male mice (*n* = 2) received intranasal treatment of 5 μl of PKH26-labeled MSC-exo and were sacrificed 24 h post administration. Mice were perfused and fixated with PBS and 4% paraformaldehyde (PFA). The brains were incubated in PFA for 24 h followed by 30% sucrose for 48 h and stored at 4 °C. The brains were frozen in chilled 2-methylbutane (Sigma-Aldrich), stored at 4 °C and subsequently sectioned into slices at 10 lm. Slides were incubated with blocking solution (5% goat/ donkey serum, 1% BSA, 0.5% Triton X-100 in PBS) for 1 h. Thereafter, slides were incubated overnight at 4 °C with primary antibody in blocking solution (mouse anti-CD11b, 1:500, Abcam) and secondary antibody in blocking solution (goat anti-mouse Alexa 488, 1:500, Molecular Probes, Invitrogen) for 1–2 h at room temperature. Next, nuclei were counterstained with DAPI (1:500; Sigma-Aldrich). Sections were ultimately mounted with fluorescent mounting solution (Fluoromount-G, Southern Biotech), covered with a cover slide, and sealed with nail polish.

#### Brain sample dissection

MSC-exo and saline-treated Shank3B KO (*n* = 5) and WT (*n* = 5) brain samples were removed from mice that had not been subjected to any behavioral testing and were kept at normal light cycle facilities (not reverse light cycle). The entire mouse brain was removed at approximately 12:00 pm (light cycle is 7:00 am to 7:00 pm) and placed in an adult mouse brain matrix (Zivic Industries, Pittsburgh, USA). Brain slices (bregma 2.8–1.42) were removed, and prefrontal cortex regions were obtained by using a 13-gauge biopsy punch needle (VGC, New Delhi, India). The cerebellum was removed using a scalpel. Brain samples were frozen with dry ice and kept in − 80 °C until mRNA extraction.

### RNA analysis

Real-time polymerase chain reaction (PCR) was performed on an ABI ViiA™ 7 RealTime PCR detection system in 10-μl volume-containing FastStart Universal SYBR Green Master (Roche, Basel, Switzerland) and primers (Supplementary Table 1) at a concentration of 0.5 μM each. Ten nanograms of cDNA was dispersed in each well, and all samples were tested in triplicates. The PCR program consists of a 15-min activation phase at 95 °C, followed by 40 cycles at the following temperatures: 10 s of 94°, 30 s of 60°. Real-time PCR data were normalized to the housekeeping gene HPRT.

### Statistical analysis

All behavioral and molecular experiments were analyzed with GraphPad (Prism). UV was analyzed by SASLab Pro (Avisoft). One-way ANOVA followed by Bonferroni correction was used for social behavioral tests and UV. Power calculation for the real-time PCR was calculated with an online calculator, (http://onlinestatbook.com/2/calculators/power_calc.html). The two-tailed power value was 0.985 which met out requirement for the five samples. One-way ANOVA followed by Tukey’s post hoc was used for real-time PCR analysis.

## Results

### Characterization of MSC-exo

**Fig. 1:**
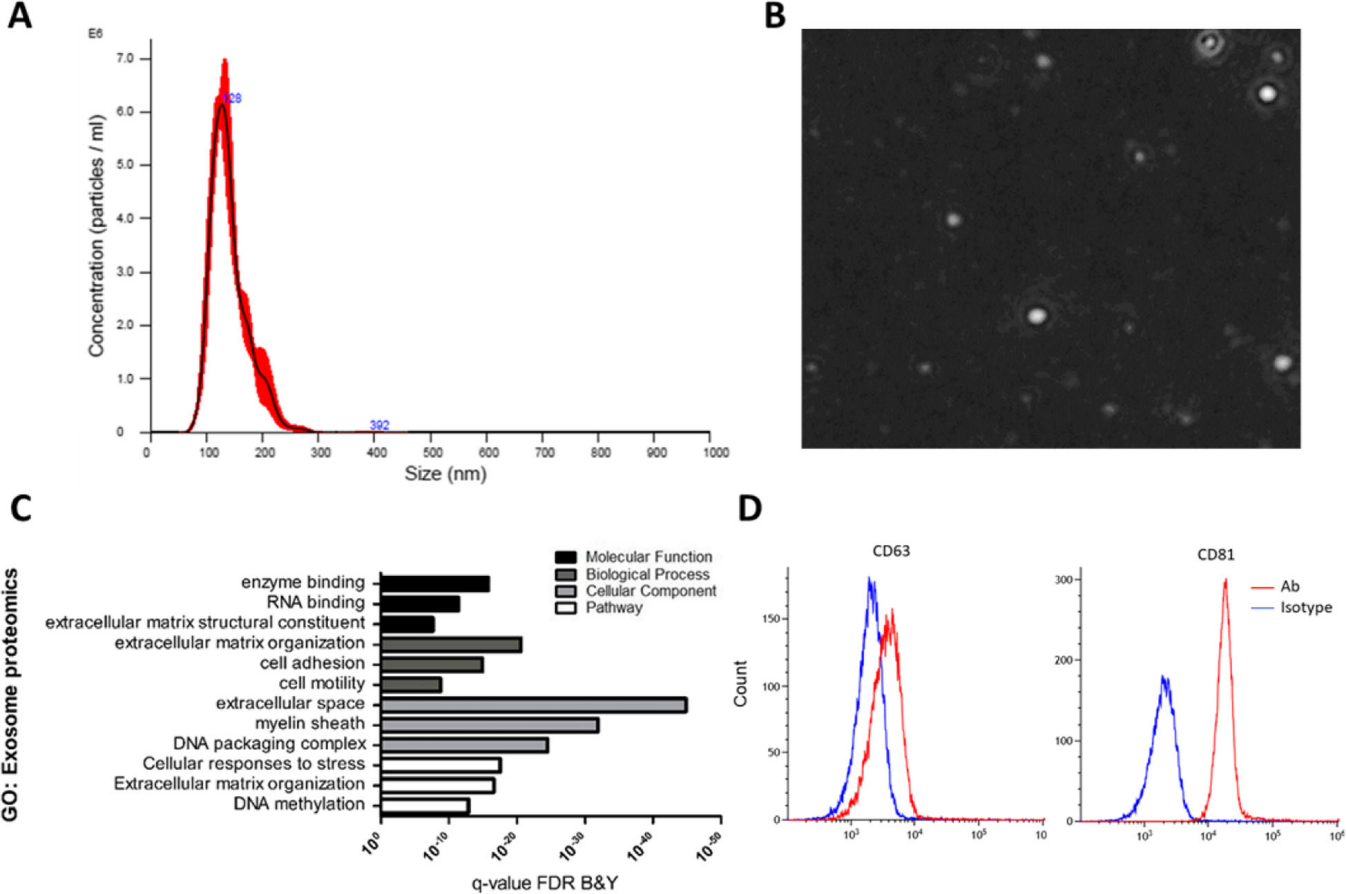
Characterization of MSC-exo by nanosight and bead-coated flow cytometry. **a** Concentration and size distribution of MSC-exo. **b** Visualization of MSC-exo by nanosight. **c** Gene ontology of the exosomal proteomics content. **d** FACS analysis of exosome expression of surface molecules; 50 μl of exosomes were incubated with 12.5 μl of 4-μm-diameter aldehyde/sulfate latex beads and stained with CD63-APC or CD81-APC Abs (red lines) or negative control IgG1 Isotype Ab (blue line)

The characterization of MSC-exo was done by nanoparticle tracing analysis technology (NTA) using Nanosight. We found that the mean exosome size is 140.5 ± 2.5 nm, and the concentration is 4.05 × 10^7^ ± 3.26 × 10^6^ particles/μl (Fig. 1 a, b)_._ In addition, we observed positive expression of essential surface molecule markers of exosomes by flow cytometry analysis for CD81 and CD63 using aldehyde/sulfate latex beads (Fig. 1d). To further characterize the protein capacity of the MSC-exo, we performed proteomics analysis, followed by gene ontology (GO) analysis. Enriched terms include enzyme binding, extracellular matrix, organization, extracellular space, and the cellular response to stress pathway (Fig. 1c).

#### MSC-exo treatment leads to significant behavioral improvement of Shank3B KO autistic-like phenotypes

To test if MSC-exo affects ASD-like behavior in Shank3B KO mice, we administered MSC-exo intranasally at 4 weeks of age, and 3 weeks post treatment-performed behavioral assays. Shank3B mice behavior was compared to WT littermates to ascertain they display expected behavioral phenotypes, as well as to evaluate treatment robustness and efficiency of MSC-exo treatment.

**Fig. 2:**
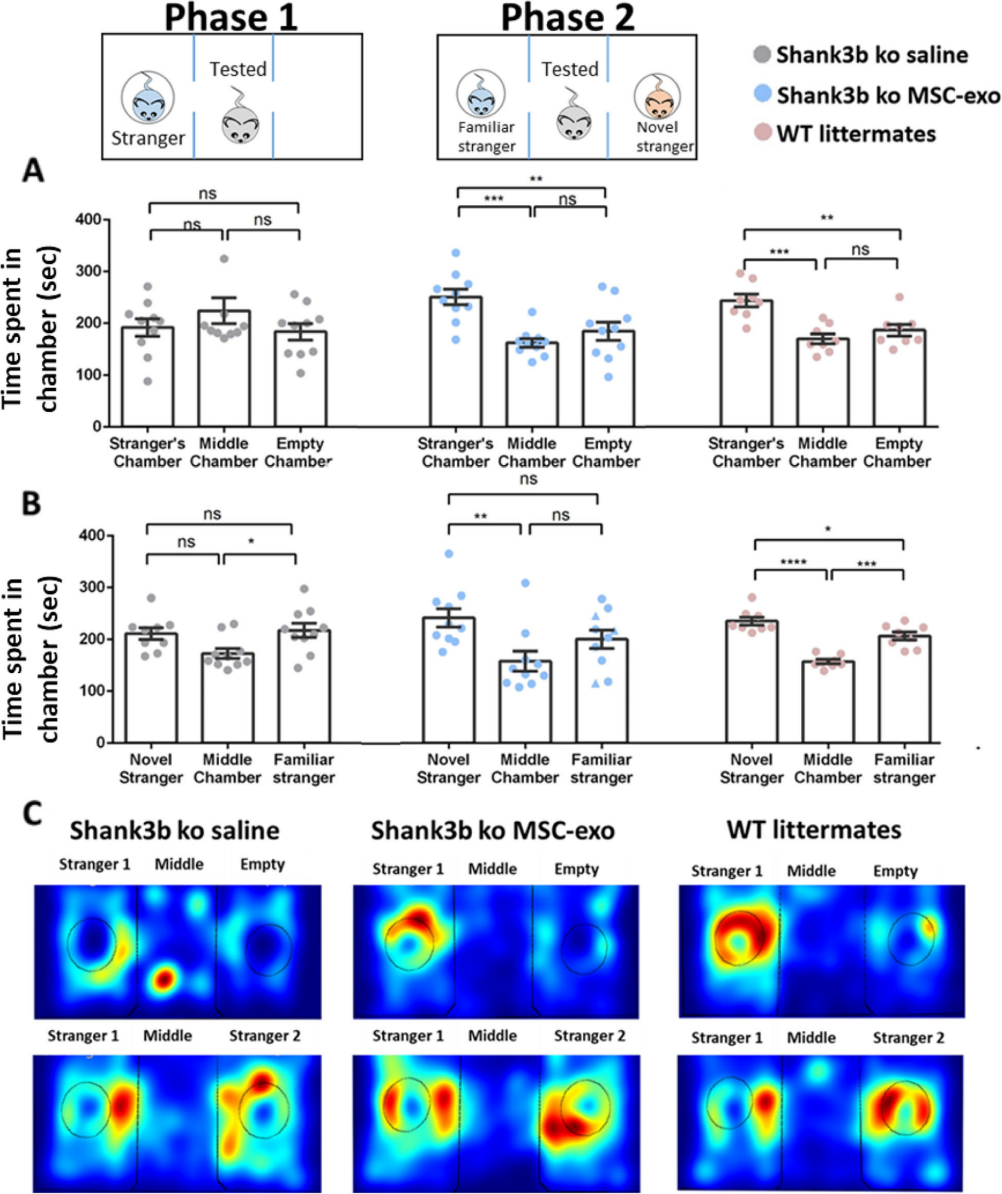
Intranasal treatment of MSC-derived exosomes rescue social behavior in *Shank3B* KO mice in the three-chamber test. **a** In the social interaction test, MSC-exo-treated Shank3B KO mice spent more time in the chamber containing the stranger compared with the empty chamber when treated with exosomes, while Shank3B KO mice treated with PBS showed no preference. WT mice spent more time in the chamber containing the mouse compared with the empty one. **b** In the social novelty test, MSC-exo-treated Shank3B KO mice spent more time in the chamber containing the novel mouse, while Shank3B KO mice treated with PBS showed no preference. WT mice spent more time in the chamber containing the novel mouse. *p < 0.05, **p < 0.01, ***p < 0.001. **c** Representative heat map for each group (top panel—phase 1, bottom panel—phase 2). One-way ANOVA followed with Bonferroni correction was used for behavioral tests analysis. Data are presented as means ± SEM

**Fig. 3:**
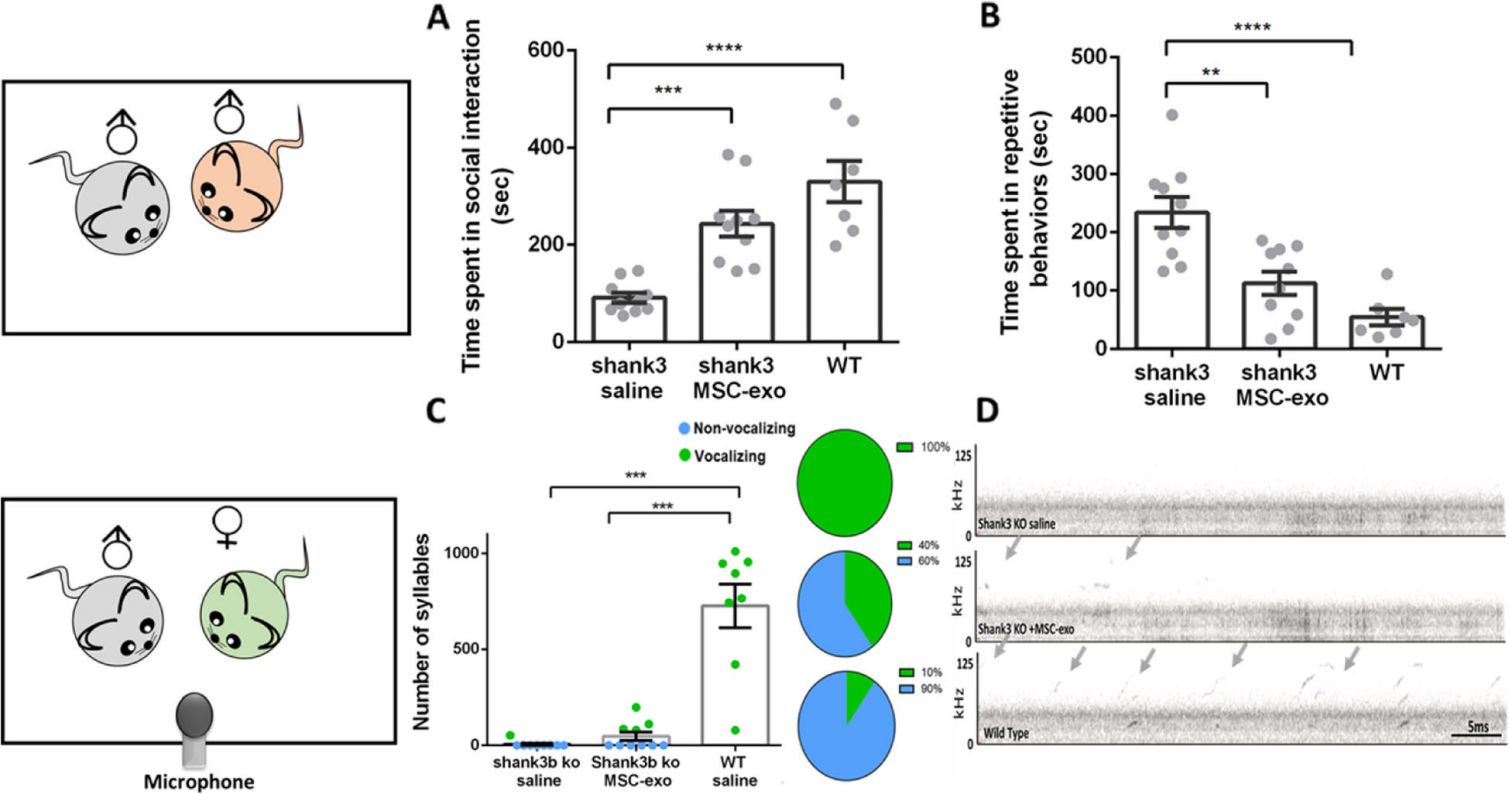
MSC-exo ameliorates the social interaction and communication autistic domains of Shank3B KO mice. **a** Male-to-male social interaction was significantly improved in the MSC-exo-treated Shank3B KO mice, compared with their saline-treated littermates. **b** Repetitive behaviors of grooming and digging were significantly reduced in the MSC-exo-treated Shank3B KO mice compared with their saline-treated littermates. **c** Though not statistically significant, MSC-exo-treated Shank3B KO mice presented more UVs compared to their saline-treated littermates. Interestingly, while all WT mice were vocalizing, only 10% of the saline-treated mice and 40% of the MSC-exo-treated mice were vocalizing. **d** Representation of the spectrogram of the vocalizations of each group. One-way ANOVA followed with Bonferroni correction was done for behavioral and vocal tests analysis. Pie charts represent the percentages of vocalizing vs non-vocalizing mice in each group. Data are presented as mean ± SEM and scatterplot. **p < 0.01, ***p < 0.001, ****p < 0.0001

Shank3B KO mice were tested for social interaction, vocal communication, and repetitive behaviors. In the social interaction domain, we used two independent tests: the three chambers (Fig. 2) and the reciprocal dyadic social interaction test (Fig. 3a). In the three chambers test, a stranger WT mouse was placed in one of the chambers, and the tested mouse could freely move between middle and side empty chambers (phase 1). MSC-exo-treated mice spent significantly more time in the chamber with the stranger mouse (251.1 ± 50.3 s) compared to the both empty chamber (185 ± 55.2 s) and the middle one (163.1 ± 26.1 s) (one-way ANOVA, F_2,27_ = 7.17, *p* < 0.01, Bonferroni). In the saline-treated Shank3B KO mice, the time they spent with the stranger (192.2 ± 52.7 s), empty (218.6 ± 78.2 s) or in the middle (187.9 ± 48.5) chamber was comparable without significant differences. In the WT littermate group, the time they spent with the stranger was significantly longer (243.5 ± 35.7 s) than in the empty (177.4 ± 26.3 s) or the middle (206.1 ± 31.9 s) chambers (one-way ANOVA, F_2,21_ = 13.217, *p* < 0.001, Bonferroni, Fig. 2a).

In the next social test, a novel stranger mouse was placed in the empty chamber and the familiar stranger mouse was left in the other chamber. The tested mouse could freely move between the chambers (phase 2). Here, MSC-exo-treated Shank3B KO mice spent significantly more time with the new stimulation mouse (241.7 ± 56.9 s) compared to the known mouse (200.4 ± 61.6 s) or the empty chambers (157.4±55.2sec). The saline treated mice presented no preference in their time spent in each chamber (194 ± 47.4 s with new stimulation 214.1 ± 52.7 s in the empty chamber and 160.8 ± 42.2 s with old stimulation). WT littermates spent significantly more time in the chamber with the new stranger (243.6 ± 39.3 s) compared to the familiar mouse (206.8 ± 32.9 s) and the empty chamber (157.3 ± 29.8 s) (one-way ANOVA, F_2,21_ = 23.9, *p* < 0.001, Bonferroni, Fig. 2b). Representative heat maps for each group in each phase are shown in Fig. 2c.

In the male-to-male reciprocal dyadic social interaction test, MSC-exo-treated Shank3B KO mice spent significantly more time engaging in social interaction with a stranger male (293.2 ± 26.2) compared with their saline littermates (96.9 ± 10.1), their results were similar to their WT littermates (330.4 ± 42.2, Kruskal–Wallis test F_3,27_ = 19.20, *p* < 0.001, Fig. 3a). Repetitive behaviors during social interaction was significantly rescued in MSC-exo-treated Shank3B KO mice (112.4 ± 19.9) as compared to their saline-treated littermates (234.2 ± 26.2) and was comparable to the WT littermates (54.9 ± 11.5, Kruskal–Wallis test F_3,27_ = 15.5, *p* < 0.001, Fig. 3b).

In the behavioral male-to-female ultrasonic vocalizations test, we found no significant differences between MSC-exo-treated mice and saline-treated Shank3B KO mice in the number of syllables. Yet, since most of the saline-treated Shank3B KO mice did not produce any syllables and some of the MSC-exo mice did, we quantified the difference in percentages. Interestingly, while all WT mice were vocalizing, only 10% of the saline-treated mice and 40% of the MSC-exo-treated mice show vocalization (Fig. 3c, d).

#### MSC-exo cross the blood–brain barrier after intranasal administration and accumulate in the cortex and cerebellum

**Fig. 4:**
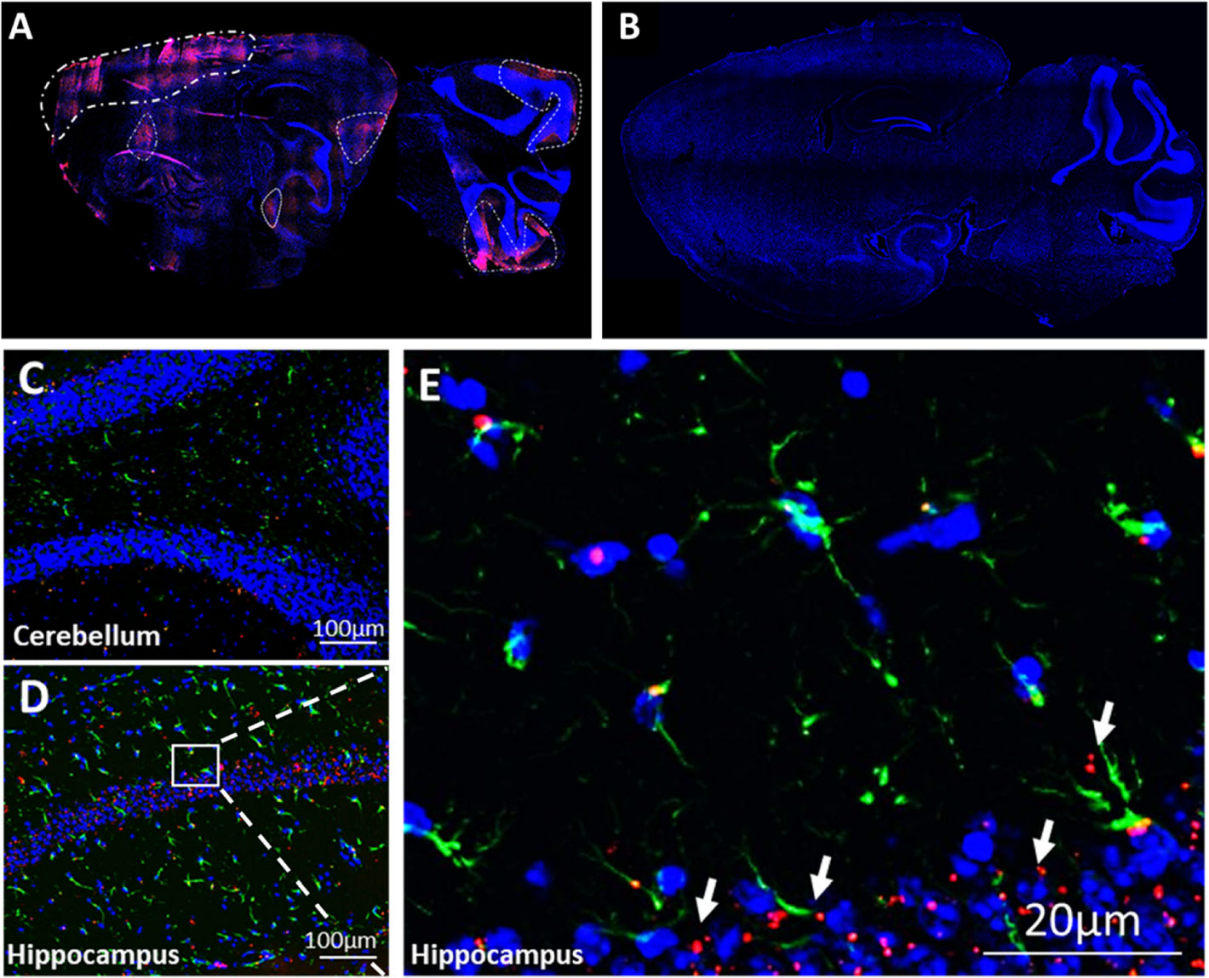
MSC-exo can cross the BBB and integrate into the cells in the tissue. **a** Complete sagittal section of Shank3B KO shows MSC-exo are found in the parenchyma and accumulate mainly in the area of the cortex, cerebellum, and some accumulation in the hippocampus (96 h post intranasal administration). **b** Complete sagittal section of WT shows complete evacuation of MSC-exo from the brain (96 h post intranasal administration). **c**, **d** Magnification of the cerebellum and hippocampus tissues of Shank3B KO shows MSC-exo are found in the tissue. **e** Magnification of the CA1 area with DAPI (blue), PKH26 exosomes (red), and astrocytes (GFAP green)

We have previously shown that MSC-exo migrate to damaged tissues in the brain after intranasal administration [15–17]. In the BTBR mouse model of autism, we saw MSC-exo accumulating in the areas of the frontal cortex and the cerebellum, while in WT C57BL/6J mice, we could not detect any accumulation, and the MSC-exo evacuated out of the brain within 24 h. Furthermore, our previous data suggest that this migration and accumulation pattern is associated with inflammation. MSC-exo accumulated in the cortex and cerebellum areas of Shank3B KO mice, and a small accumulation in the hippocampus was also observed. In the brains of WT littermates, MSC-exo were completely evacuated without any traces, as expected (Fig. 4).

### Increased inhibitory GABA-Rb1 receptors in the frontal cortex

Previous observations in patients diagnosed with autism and studies in mouse models raised the theory that excitation/inhibition imbalance takes part in ASD’s neuropathology [37]. Other studies revealed that oxytocin signaling is disrupted in ASD, and oxytocin treatment in Shank3B KO rats demonstrated improved behavioral and neurophysiological phenotypes [43]. Additionally, a previous study from our lab revealed that treating the Shank3B KO mouse with the bacteria *Lactobacillus reuteri* increases GABA receptor expression and increases oxytocin levels, which results in rescued behavioral phenotypes [44]. To test whether MSC-exo treatment will have a similar effect, we looked for possible changes in gene expression in several key GABAergic receptors and oxytocin.

**Fig. 5:**
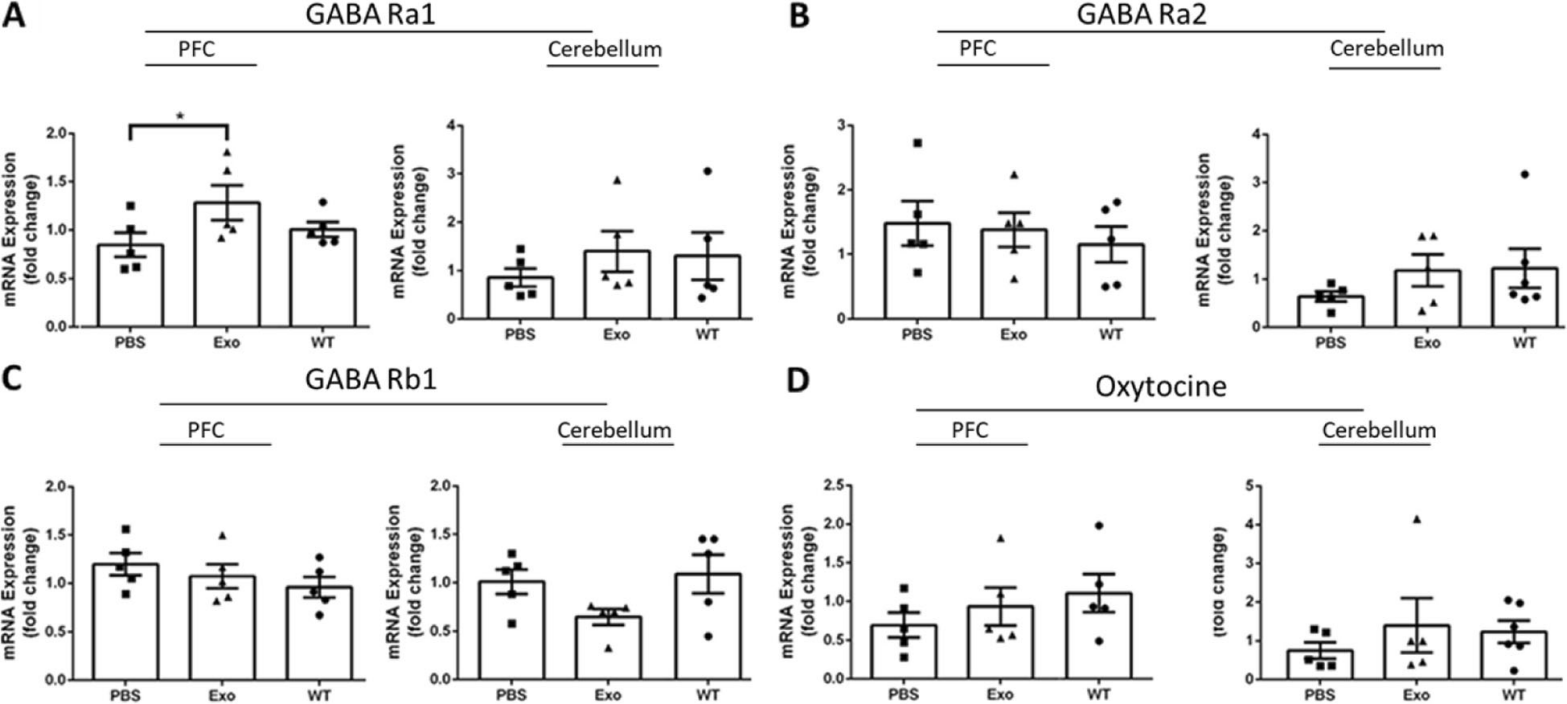
Higher expression of GABA Ra1 were observed in the PFC of MSC-exo-treated mice, yet GABA Ra2, GABA Rb1 and oxytocin remained unchanged in the PFC and the cerebellum. **a–d** GABA Rb1 expression was significantly increased in the PFC but not in the cerebellum (**a**). There was no significant difference in GABA-Ra2 (**b**) and GABA-Rb1 (**c**). Also, there was no difference in oxytocin receptors (**d**). One-way ANOVA followed with Tukey’s post hoc was done for the rtPCR analysis. *p < 0.05. Data are presented as means. Error bars represent the ±SEM

Although no noteworthy difference was found in RNA levels of GABA Ra1, GABA Ra2 and oxytocin in the frontal cortex and cerebellum, we observed a significant increase in GABA Rb1 RNA in the prefrontal cortex (PFC) after MSC-exo treatment in Shank3B KO mice (one-way ANOVA, F_2,15_ = 6.5, *p* < 0,05, Fig. 5a–d). This result, though minor, may indicate inhibitory upregulation in the PFC after MSC-exo treatment. Importantly, RNA levels of inflammatory markers such as - TNFα**,** IBA1, and IL1 were also tested and were not found significantly altered between the groups (figure S1).

## Discussion

In this study, we show that intranasally administered MSC-exo can ameliorate several ASD-like behaviors in the Shank3B KO model including the social and communicational phenotypes. Additionally, we show that exosomes migrate to several areas of the mouse brain including the PFC and cerebellum which have previously been implicated in ASD pathology. Finally, we observed an increase of GABA-Rb1 in the PFC in Shank3B KO mice treated with MSC-exo.

The Shank3B KO mice have previously shown multiple deficits in social interaction, UVs, and repetitive behaviors [29, 30]. Furthermore, Mei et al. (2016) have shown that replacing the Shank3B variant with the intact gene leads to a recuse of the behavioral autistic-like deficits [45]. In this study, we attempted to reverse ASD-like behaviors in an approach we formerly demonstrated to be successful in the BTBR model, and so has potential to be used therapeutically as it does not involve any invasive actions.

Shank3B KO mice were treated with MSC-exo according to the administration protocol used in our previous BTBR study, and their behaviors were compared with their WT littermates and Shank3B KO littermates that were treated with saline. We found significant improvement in social interaction in Shank3B KO mice treated with MSC-exo in independent tests: the dyadic reciprocal social interaction and three-chamber social test for social preference. In the dyadic reciprocal social interaction, the MSC-exo Shank3B KO-treated group spent significantly more time engaging in social interaction with a stranger mouse, compared to their saline treated littermates. We further confirmed this result using the three-chamber test. Shank3B KO mice treated with MSC-exo presented a clear preference to the chamber with the stranger mouse and the novel mouse compared to the empty chamber and familiar mouse, respectively. Thus, our data indicate that MSC-exo treatment can benefit the social interaction domain of the ASD-like behaviors of the Shank3B KO model.

In the UV test, we characterize the vocal communication domain and found no significant improvement in the number of syllables made by the Shank3B KO mice treated with MSC-exo and saline groups. However, in the current study, we noticed that while all the WT mice performed UVs, only 10% of the saline-treated Shank3B KO group performed UVs at all, which suggests that this genetic variation has a severe influence on UVs. To better understand if exosome treatment has any effect on UVs in this model, we questioned if there was any UV post MSC-exo treatment and found that 40% of the mice performed at least one UV. This result implies that exosome treatment may improve unique aspects of vocal communication in Shank3B KO mice and should be further investigated.

In the repetitive behaviors domain, grooming and digging during social interaction were measured. A significant difference was found between saline and the MSC-exo-treated group. MSC-exo-treated mice spent significantly less time in self-grooming and digging and more in social interaction.

Altogether, herein, we report that MSC-exo intranasal treatment can lead to significant behavioral amelioration in the social interaction and vocal communication domains of Shank3B KO mice. We find that these results are interesting as we have shown significant improvements in social interaction and grooming in the BTBR model upon MSC-exo administration [9, 46]. In the UV test, MSC-exo treatment was not as beneficial for the Shank3B KO as it was for the BTBR and so suggests a different mechanism of effect.

We have also demonstrated that MSC-exo tend to migrate to the frontal cortex and cerebellum. This tendency was pathology-specific and was tested in other mouse models. In a stroke model induced by injection of endothelin-1, the MSC-exo selectively targets the damaged area, while in other pathologies such as AD models of transgenic mice (5xFAD), they were found mainly in regions of the hippocampus. Interestingly, in WT mice, the MSC-exo could not be detected in the brain 24 h post the intranasal administration [15, 16]. Using the same rationale, we examined the migration and neuro-distribution pattern of the MSC-exo in the Shank3B KO mice. We found that 96 h post intranasal administration, a complete evacuation of MSC-exo was observed in the WT brains compared to significant accumulation in the frontal cortex and cerebellum in the Shank3B KO brains. It is of note that some accumulation was also found in the area of the hippocampus and medial entorhinal cortex. These findings comply with our previous results spotlighting the specificity of exosome migration to neuropathological tissues in different pathologies. This ability to target and accumulate in particular pathological regions of the brain may overcome the lack of specificity current treatments offer, which result in multiple adverse effects, and may even offer a novel method for diagnostics.

In order to better understand the molecular influence of MSC-exo on Shank3B KO mice, we first measured gene expression of inflammatory markers including TNFα, IBA1, and IL1. This was under the assumption that MSC-exo may lead to the reduction of inflammation in the damaged tissues, thus contributing to behavioral amelioration [42–44]. Yet, we found no evidence of inflammatory suppression led by the MSC-exo. Previous studies in an AD model suggested that cognitive deficits rose due to inflamed blood–brain barrier (BBB), which was rescued post exosome treatment [47]. Based on our knowledge, the pathological and phenotypical abnormalities of the Shank3B KO mice are not caused by damages to the BBB but by the genetic mutation in the protein leading to synaptic dysfunction. Since it is known that exosomes can cross the BBB also in healthy mice, we do not assume that the behavioral amelioration of the treated Shank3B KO mice is caused by improvement of the BBB but rather by molecular and protein changes in the neurons caused by the natural capacity that the MSC-exo introduced to the damaged cells.

We also expected a reduction in oxytocin expression in accordance with the ASD hypothesis [48, 49]. Nevertheless, we could not find supporting evidence that links MSC-exo treatment to alternations in the expression levels of oxytocin in both the PFC and the cerebellum.

Another approach regarding the neurological changes in autistic brains followed by genetic mutation refers to the excitation–inhibition imbalance found in postmortem analysis of autistic brains and was supported by animal models [37, 50]. In addition, we observed a decrease in GABA receptor subunits in the Shank3B hippocampus in a previous study published by our group [44]. In that study, treatment of Shank3B KO mice with *L. reutri* increased GABA receptor subunit expression and resulted in the improvement of behavioral deficits. Therefore, we measured the expression of GABA subunits GABA Ra1, GABA Ra2, and GABA Rb1 in the prefrontal cortex and cerebellum. We found a significant increase in expression in GABA Ra1 in PFC of the treated mice compared to the saline group. The levels of GABA Ra1 in the cerebellum as well as other GABA subunits remained unchanged. This result suggests the involvement of GABA-mediated pathways in the prefrontal cortex may be attenuated by MSC-exo treatment which may contribute to improvement in behavioral deficits observed in ASD. *SHANK3* is a scaffolding protein that plays a crucial role in anchoring NMDA and AMPA receptors to the postsynaptic membrane. Therefore, future studies should include evaluating if MSC-exo treatment affects these receptors as well as part of the excitation–inhibition imbalance theory of ASD.

In recent years, there has been a growing interest in research aiming to find common molecular and physiological deficits in multiple ASD mouse models that could be targeted pharmaceutically [51–53]. We have demonstrated that MSC-exo treatment had a significant effect on all the core ASD-like behaviors of the autistic-like behaviors of two different mouse models.

Social interaction and ultrasonic communication require high-level synchronization of sensory input and behavioral output. Sensory integration and coordination deficits have been suggested to be one of the underlying mechanisms of the ASD patients [54–56]. Stem cell therapy has been previously used on ASD children with long-term beneficial effects [57]. Bone marrow MSC transplantation has been proven to be safe to use in several clinical trials [58–60]. Although it is clear that MSCs have beneficial properties that can be used safely for clinical purposes, recent evidence shows that the therapeutic effect of MSCs is largely mediated via the secretion of exosomes that contain important molecular information [35, 61]. This study supports this claim; however, further investigation into the molecular processes by which exosomes communicate with brain cell populations is required to elucidate its efficacy.

### Limitations

Although the behavioral scoring of the MSC-exo-treated group was significantly improved compared to their saline-treated littermates, there is much more to learn regarding the mechanism of action of the MSC-exo.

## Conclusions

Altogether, our data suggest that that MSC-exo may be efficient to treat ASD symptoms caused by a specific genetic mutation. This finding is extremely relevant for clinical indications since 1–2% of ASD patients carries specific mutation in the *SHANK3* gene.

## Supplementary information

**Additional file 1:** Table S1

**Additional file 2: **Figure S1. rtPCR for inflammatory markers shows no significant difference between the groups. **A.** TNFα **B**. IBA1 **C.** IL1. One-way ANOVA followed with Tukey’s post hoc was done for the rtPCR analysis. Data is presented as means Error bars represent the ±S.E.M.

## Data Availability

The datasets used and/or analyzed during the current study are available from the corresponding author on reasonable request.

## Abbreviations

ASD: Autism spectrum disorders
Shank3B KO: Knock out of the B domain in the *SHANK3* gene by deletion of the 22q13.3 location
BTBR: BTBR T+tf/J
MSC: Mesenchymal stem cells
MSC-exo: Mesenchymal stem cell-derived exosomes
miRNA: micro RNA
PSD: Postsynaptic density protein
GABA Ra1/ GABA Ra2/ GABA Rb1: GABA receptor's subtypes a1, a2, and b1
