# Supplementary figures and images for: Exosomes derived from mesenchymal stem cells improved core symptoms of genetically modified mouse model of autism Shank3B
Source: Mol Autism. 2020 Aug 17;11:65. doi: 10.1186/s13229-020-00366-x (PMC7433169; doi:10.1186/s13229-020-00366-x)

**
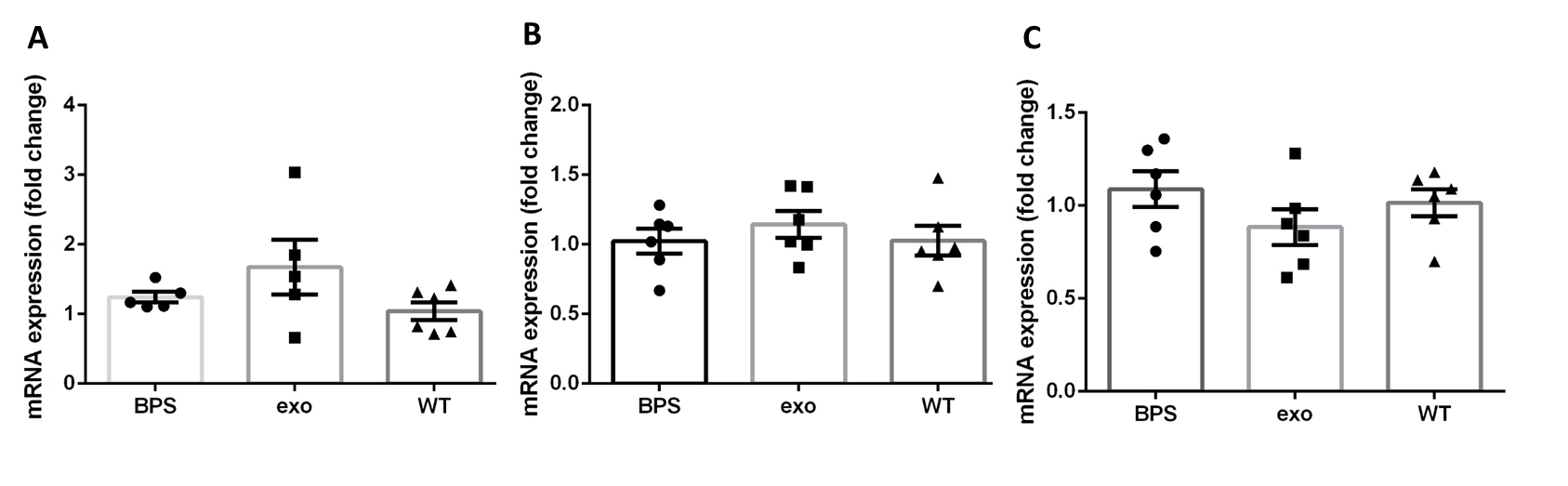
**

Supplement: Supplementary file 2 — Additional file 2: Figure S1. rtPCR for inflammatory markers shows no significant difference between the groups. A. TNFα B. IBA1 C. IL1. One-way ANOVA followed with Tukey’s post hoc was done for the rtPCR analysis. Data is presented as means Error bars represent the ±S.E.M. [file 13229_2020_366_MOESM2_ESM.docx]
